# Supplementary material for: Aurora A-dependent CENP-A phosphorylation at inner centromeres protects bioriented chromosomes against cohesion fatigue
Source: Nat Commun. 2018 May 14;9:1888. doi: 10.1038/s41467-018-04089-9 (PMC5951908; doi:10.1038/s41467-018-04089-9)
Supplement: Supplementary file 1 — Supplementary Information [file 41467_2018_4089_MOESM1_ESM.docx]

**SUPPLEMENTARY INFORMATION**

**Aurora A-Dependent CENP-A Phosphorylation at Inner Centromeres Protects Bioriented Chromosomes Against Cohesion Fatigue**

Eot-Houllier et al.


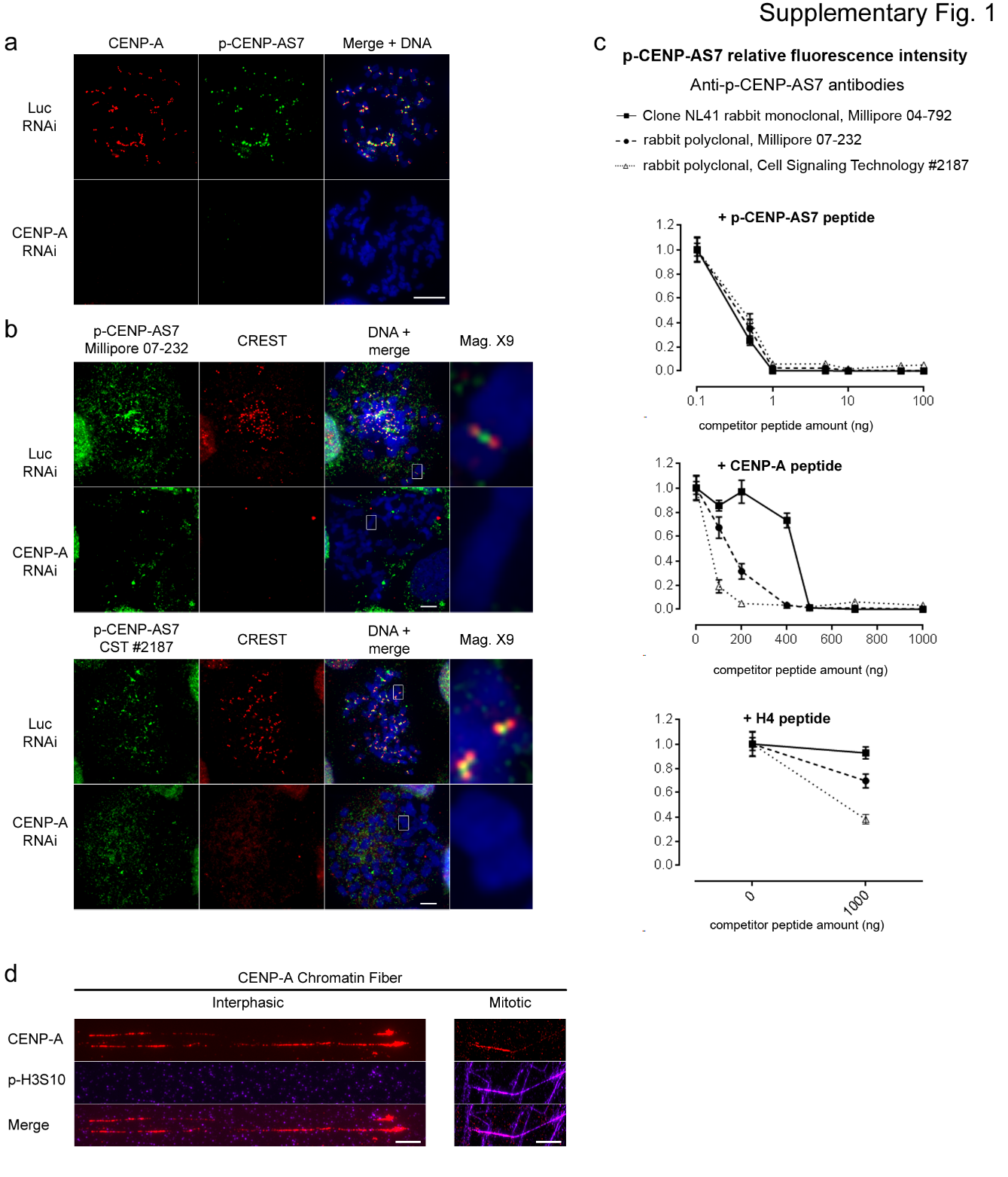


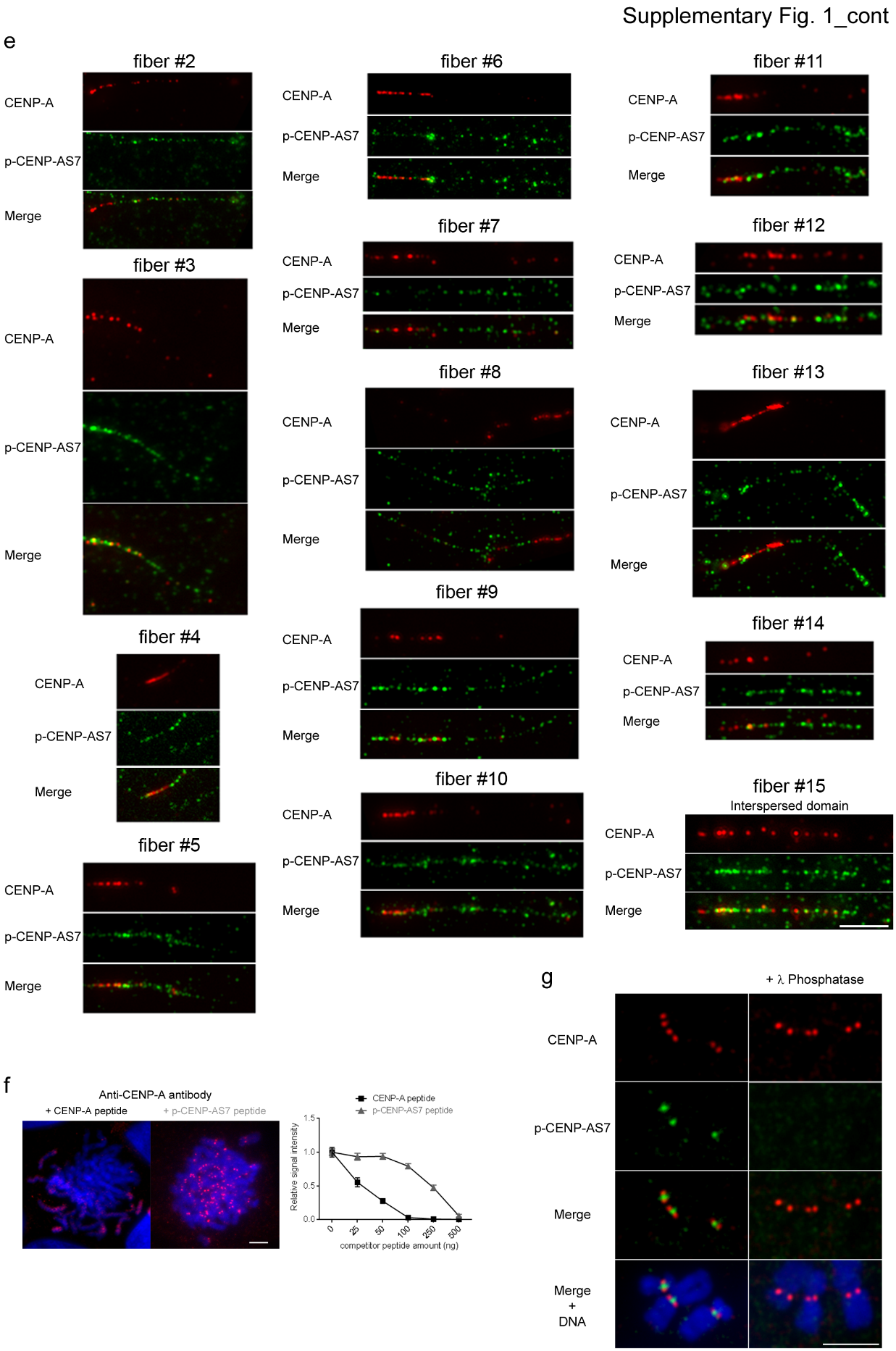


**Supplementary Figure 1.** (A) Metaphase chromosome spreads from HeLa-S3 cells were double-stained with anti-CENP-A (red) and anti-p-CENP-AS7 (green) antibodies, before (upper panel) or after (lower panel) CENP-A depletion by siRNA. Scale bar = 10 µm. (B) Serine 7-phosphorylated CENP-A (p-CENP-AS7) was also shown, with two other commercial antibodies, to localize to the inner side of centromeres. Metaphase chromosome spreads from HeLa-S3 cells were double-stained with anti-CREST (red) and anti-p-CENP-AS7 (green) antibodies, before or after CENP-A depletion by siRNA. The results obtained with the 07-232 antibody from Millipore and the #2187 antibody from Cell Signaling Technologies are presented on the upper and lower panels, respectively. Scale bar = 10 µm. Magnifications (X9) of single chromosomes are shown. (C) Specificity of the various commercial anti-p-CENP-AS7 antibodies. Before their incubation with metaphase chromosomes for immunofluorescence studies, the abovementioned p-CENP-AS7 antibodies were incubated with various amounts (as indicated) of a serine-7-phosphorylated peptide (top graph), an unmodified N-terminal-CENP-A peptide (middle graph) or an irrelevant peptide corresponding to the N-terminal part of histone H4 (bottom graph). A quantification of the fluorescence signal intensity relative to CREST is presented for each competitor peptide. Each point correspond to the mean ± SEM (*n*>10 cells). (D) Extended chromatin fiber from interphase or mitotic HeLa-S3 cells: fibers were stained with anti-CENP-A (red) and anti-p-H3S10 (purple) antibodies. The absence or presence of p-H3S10 staining indicates the interphase or mitotic state, respectively, of the observed chromatin. Scale bar = 5 µm. (E) Mitotic stretched chromatin fibers. Extended chromatin fibers were prepared from HeLa-S3 cells blocked in mitosis by overnight nocodazole treatment. Fibers were stained with anti-CENP-A (red) and anti-p-CENP-AS7 (green) antibodies. Scale bar = 5 µm. Fibers #2 to # 14 show distinct domains of the phosphorylated and non-phosphorylated forms of CENP-A. Only fiber #15 present interspersed CENP-A and p-CENP-AS7 domains. (F) Specificity of the anti-CENP-A antibody. Before its incubation with metaphase chromosomes for immunofluorescence studies, the CENP-A antibody was incubated with various amounts (as indicated) of an unmodified N-terminal-CENP-A peptide (squares) or a serine-7-phosphorylated peptide (triangles). The peptides used were identical in sequence and length to the epitopes used to obtain the antibodies. Examples of chromosome spreads immunostained with anti-CENP-A antibodies (red) incubated with either 100 ng of CENP-A or p-CENP-AS7 peptides are shown on the left. Scale bar = 10 µm. Quantifications of fluorescence signals for the CENP-A antibody incubated with different amounts of peptide are presented on the right. Each point correspond to the mean ± SEM. (*n*>10 cells). (G) Lambda phosphatase treatment. Fixed chromosome spreads were incubated with five units of Lambda phosphatase for two hours before double-immunostaining with anti-CENP-A (red) and anti-p-CENP-AS7 (green) antibodies. Scale bar = 4 µm. Note that p-CENP-AS7 staining disappeared after phosphatase treatment, but immunostaining detected no unphosphorylated CENP-A on the inner side of the centromere.


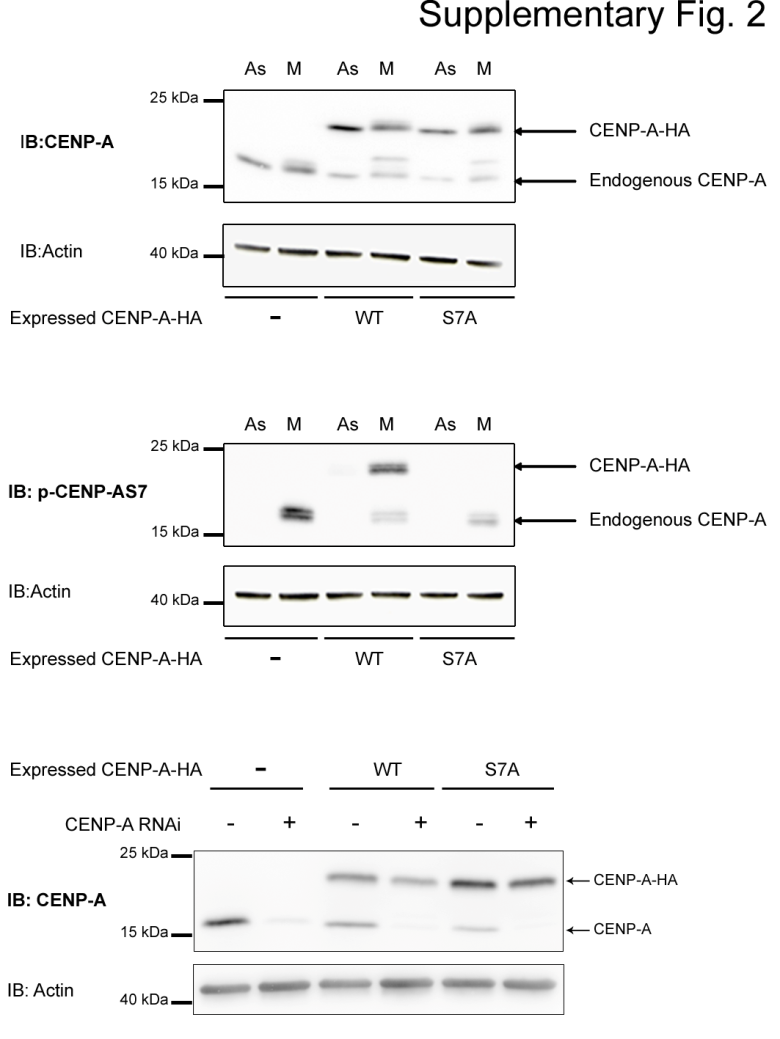


**Supplementary Figure 2**. Characterization of HeLa-S3 cell lines stably expressing CENP-A protein HA-tagged at the C-terminus. The CENP-A-WT-HA and CENP-A-S7A-HA cell lines were derived from HeLa-S3 cells stably transfected with a 3'-HA-tagged-CENP-A expression vector (CENP-A WT-HA) or a 3'-HA-tagged-CENP-A-S7A expression vector (CENP-A-S7A-HA) encoding a CENP-A protein in which the serine 7 residue was replaced by an alanine residue, preventing phosphorylation at this position. Transfected stable cell lines were cloned by limiting dilution and individual clones producing HA-tagged CENP-A in amounts similar to those for endogenous CENP-A were selected. HeLa-S3, WT-HA and A7-HA cell lines were (Mitosis) or were not (Asynchronous Cells) treated overnight with nocodazole. Protein extracts were prepared from these cells lines and immunoblotted with anti-CENP-A (upper panel) or anti-p-CENP-AS7 (middle panel) antibodies. Actin was used as a loading control. Note the absence of immunodetection with the anti-p-CENP-AS7 antibody when the serine 7 residue is replaced with an alanine residue. (Lower panel) CENP-A-WT-HA and CENP-A-S7A-HA cell lines were transfected with control or CENP-A siRNA. The corresponding cell extracts were immunoblotted with anti-CENP-A antibodies. Actin was used as a loading control. Endogenous CENP-A in HeLa-S3 cells is shown in the first lane. Note the disappearance of the endogenous CENP-A signal and the persistence of the HA-tagged-CENP-A signal, both indicated by arrows.

**
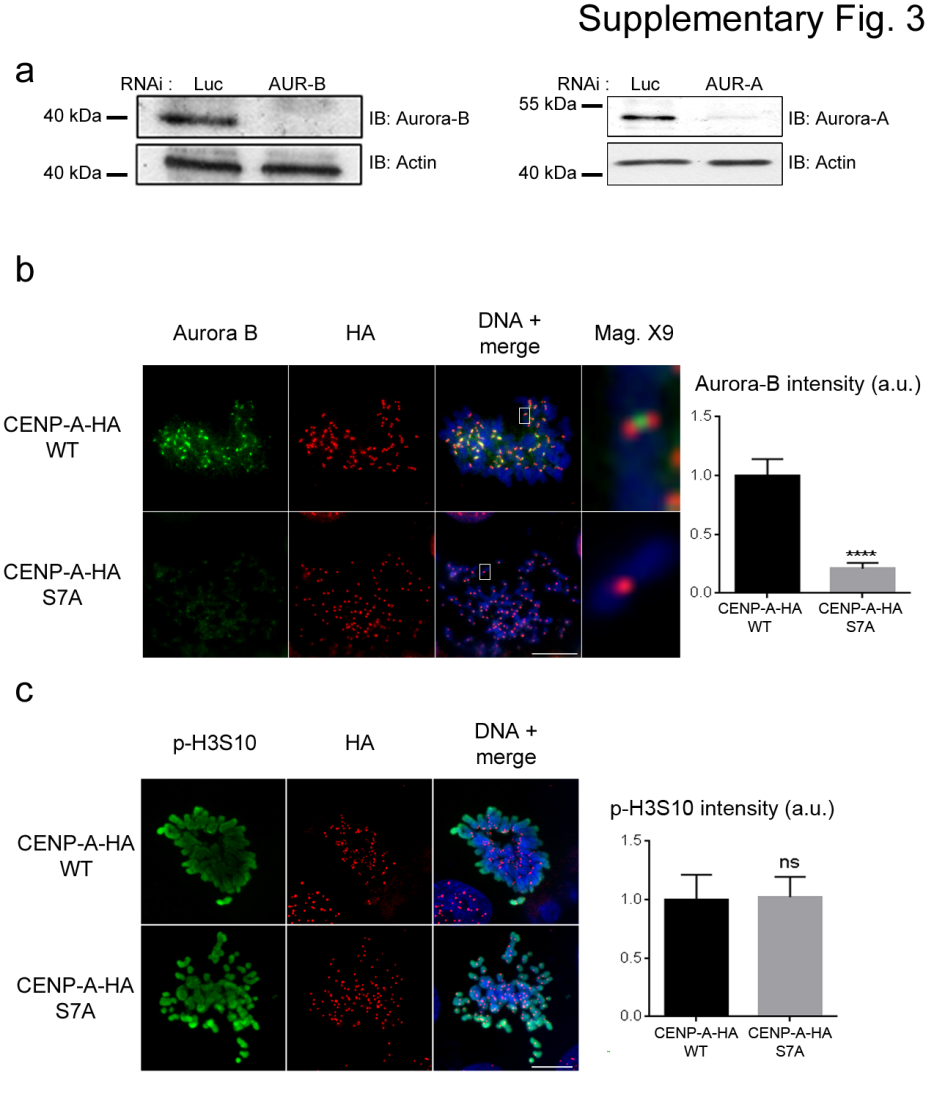
**

**Supplementary Figure 3.** Localization and activity of Aurora B after treatment with an inhibitor, mutation of CENP-AS7 or absence of CPC targeting to the centromere. (A) Aurora A and Aurora B depletion by siRNA in HeLa-S3 cells. HeLa-S3 cells were transfected with control, Aurora B or Aurora A siRNA. The corresponding cell extracts were immunoblotted with anti-Aurora B or Aurora A antibodies. Actin was used as a loading control. (B) Loss of centromeric Aurora B in the absence of phosphorylatable CENP-AS7. Chromosome spreads showing staining for HA (red) and Aurora B (green) in CENP-A-WT-HA control cells or in CENP-A-S7A-HA cells displaying sister chromatid separation. Scale bar = 10 µm. Kinetochore magnifications are shown. Quantifications of relative fluorescence intensity for Aurora B are shown (means ± SEM; *n* = 10 cells from a representative experiment; **** *P*<0.0001; two-tailed unpaired nonparametric Mann-Whitney *U* test). (C) Aurora B kinase activity is maintained in the absence of phosphorylatable CENP-AS7. p-H3S10 staining in CENP-A-WT-HA control cells or in CENP-A-S7A-HA cells displaying sister chromatid separation. Scale bar = 10 µm. The quantification of p-H3S10 fluorescence intensity is shown (means ± SEM; *n* = 10 cells from a representative experiment; ns: not significant; two-tailed unpaired nonparametric Mann-Whitney *U* test).


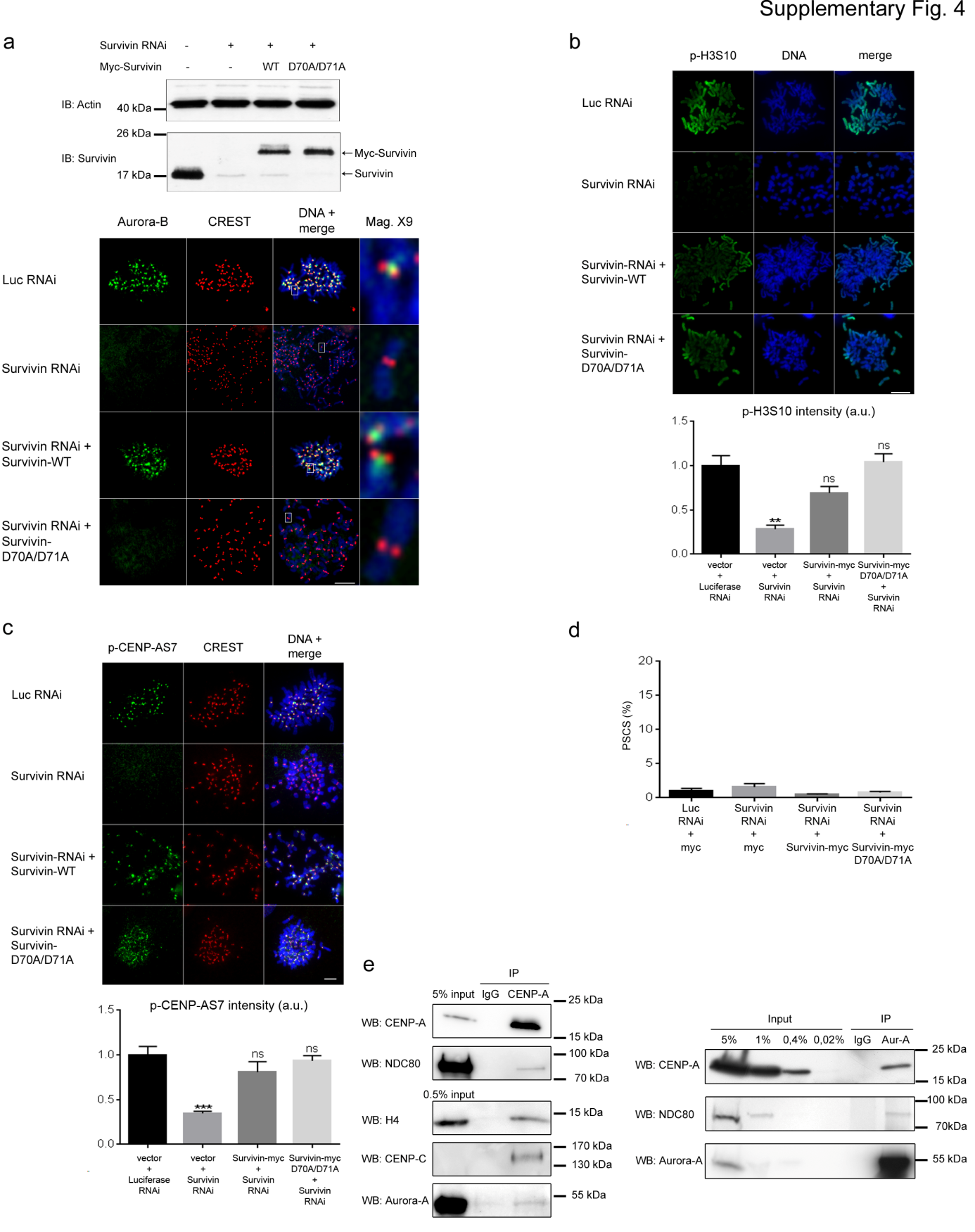


**Supplementary Figure 4**. Loss of Aurora B recruitment at the centromere does not induce premature separation of sister chromatids and endogenous Aurora A and CENP-A co-immunoprecipitate. (A) Top of the panel shows endogenous Survivin depletion in the control and Myc-tagged-Survivin (mutated or not)-expressing cell lines transfected with control or Survivin siRNA. Actin was used as a loading control. Panel bottom shows chromosome spreads stained for Aurora B (green), CREST (red) and DNA in control, Myc-tagged-Survivin WT or Myc-tagged-Survivin D70A/D71A cell lines transfected with control or Survivin siRNA. A magnification is shown for a single chromosome. Scale bar = 10 µm. (B) Chromosome spreads showing staining for p-H3S10 (green) and DNA in control, Myc-tagged-Survivin WT or Myc-tagged-Survivin D70A/D71A cell lines transfected with control or Survivin siRNA. Scale bar = 10 µm. Quantifications of fluorescence intensity for p-H3S10 are shown (means ± SEM; *n* > 15 cells from a representative experiment; ** *P*<0.01; ns: not significant; nonparametric Kruskal-Wallis test). (C) Chromosome spreads showing staining for CREST (red), p-CENP-AS7 (green) and DNA in control, Myc-tagged-Survivin WT or Myc-tagged-Survivin D70A/D71A cell lines transfected with control or Survivin siRNA. Scale bar = 5 µm. Quantifications of fluorescence intensity for p-CENP-A-S7 are shown (means ± SEM; *n* = 10 cells from a representative experiment; *** *P*<0.001; ns: not significant; nonparametric Kruskal-Wallis test). (D) The mitotic cells displaying PSCS in the mutant-survivin cell lines were quantified. Error bars correspond to the SD of the means (*n* =3 experiments). (E) Left panel: western blots of immunoprecipitated mitotic chromatin with anti-CENP-A or purified goat IgG (IP), showing the association between CENP-A and the endogenous centromeric proteins NDC80, H4 and CENP-C and endogenous Aurora A. Right panel: reverse experiment with mitotic chromatin immunoprecipitated with an anti-Aurora A or purified rabbit IgG.


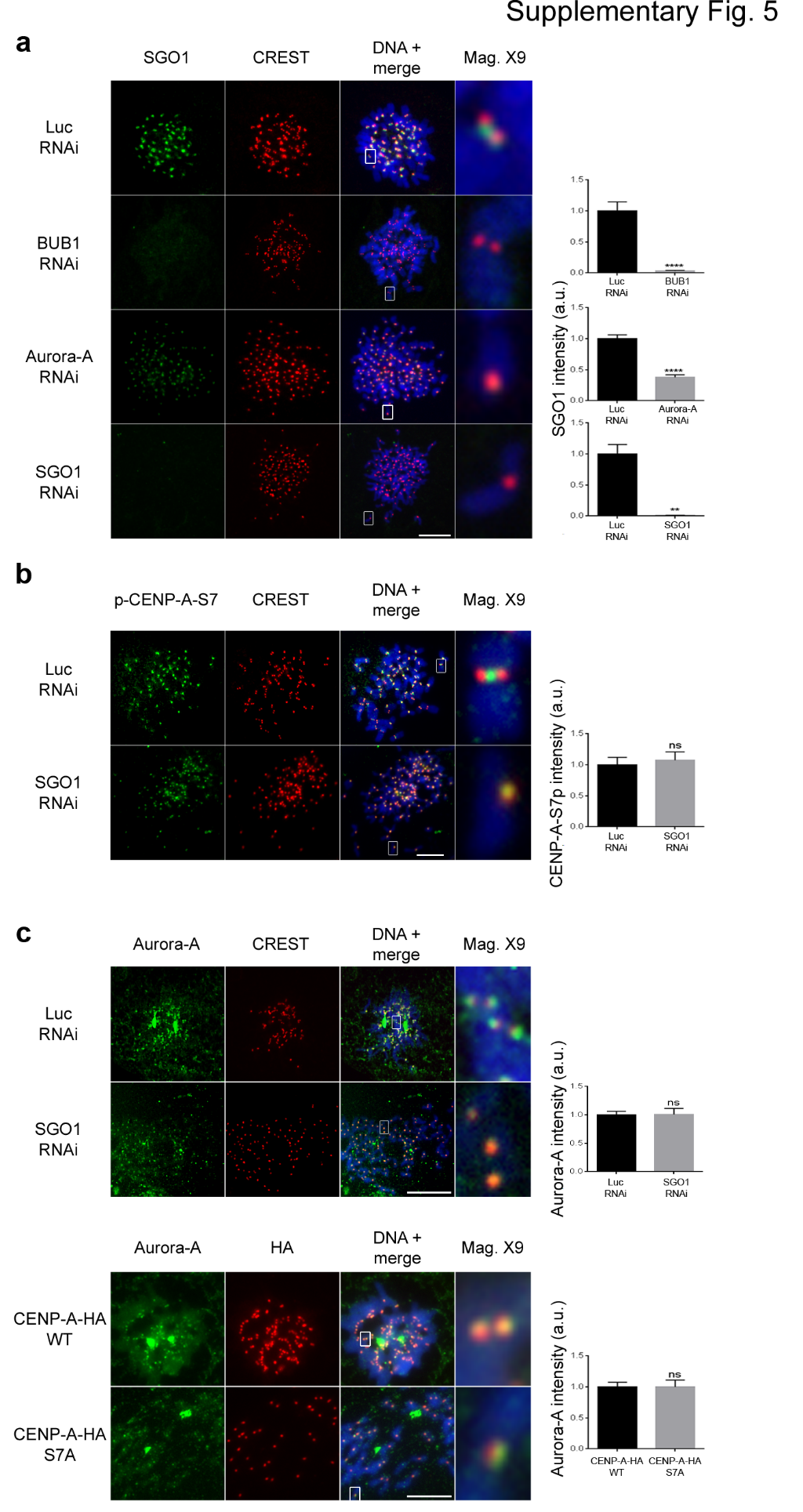


**Supplementary Figure 5**. Metaphase chromosome spreads immunostained with CREST or HA and (A) Sgo1, (B) p-CENP-AS7, (C) Aurora-A antibodies after the indicated siRNA-mediated depletions in HeLa-S3 cells or in CENP-A-WT-HA and CENP-A-S7A-HA cell lines. Scale Bar = 5 µm. Single chromosome magnifications are presented. A quantification of fluorescence intensity for each immunostaining is shown on the right. (means ± SEM; *n* >10 cells from a representative experiment; **** *P*<0.0001; *** *P*<0.001; ** *P*<0.01; ns: not significant; nonparametric two-tailed unpaired Mann-Whitney *U* test).


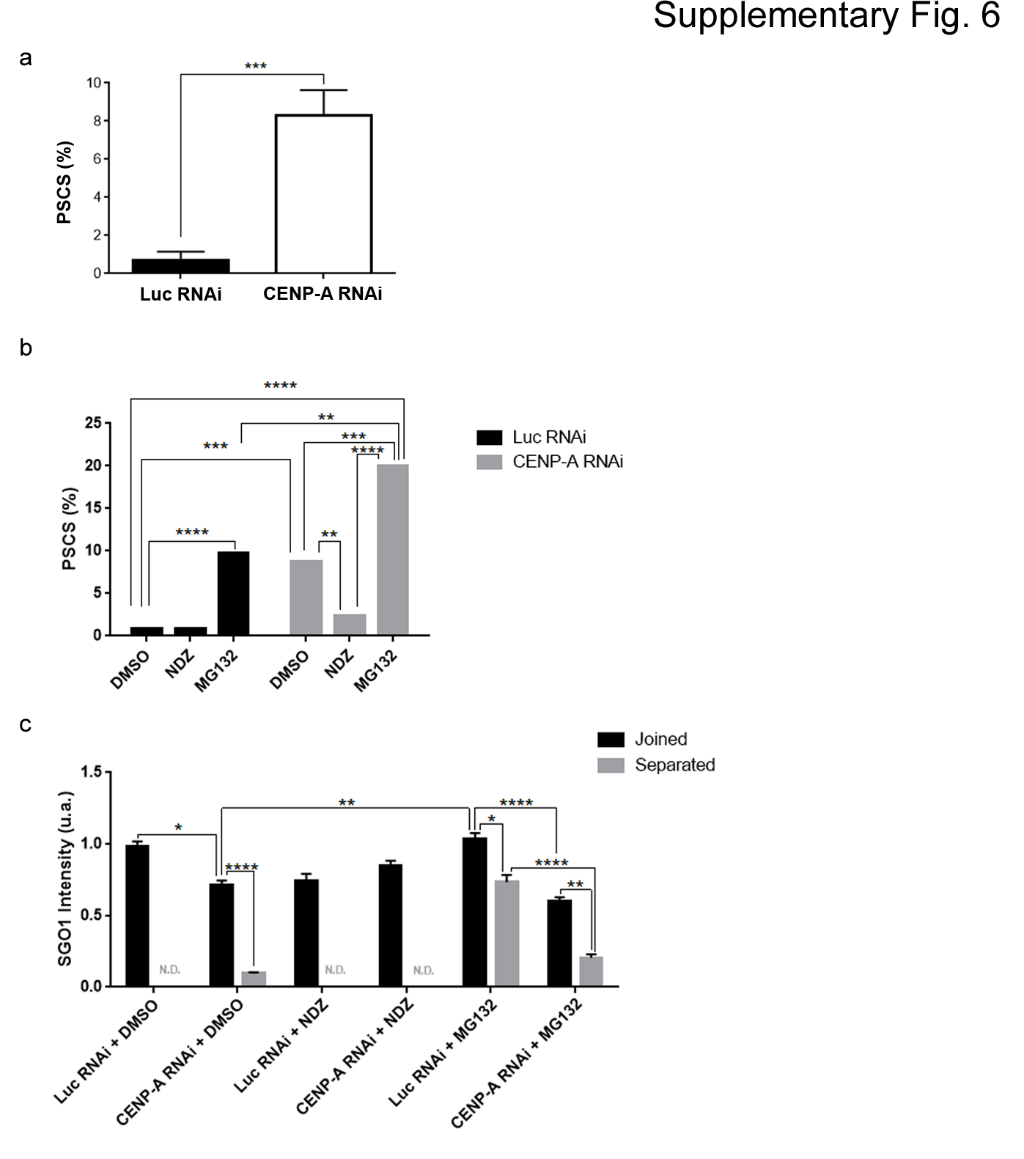


**Supplementary Figure 6**. CENP-A is required for the protection of sister chromatid cohesion during mitosis in HeLa cells. (A), Metaphase chromosome spreads were prepared 5 days following CENP-A siRNA transfection or control siRNA in the Hela S3 cell line and the PSCS were quantified. The data shown are the mean ± SD (n = 3 experiments; 200-400 cells were counted for each data point per experiment; *** *P*<0.001, one-way ANOVA). (B) & (C) 5 days following Hela S3 cells transfection with Luc or CENP-A siRNA, cells were treated with nocodazole for 3h or with MG132 for 6h before spreading of mitotic chromosomes. The data in (B) correspond to the proportion of mitotic cells displaying PSCS (n = 1 experiment; 200-250 cells were counted for each data point; **** *P*<0.0001; *** *P*<0.001; ** *P*<0.01, systematic Chi square contingency test with Yates’ correction were performed between each point with each other). (C): quantifications of fluorescence signals for Sgo1 in cell displaying joined or separated sister chromatids (SC) are shown. The graph shows the means ± SEM (*n* = 28 cells for Luc RNAi; *n* = 27 cells for CENP-A RNAi with joined SC ; *n*= 23 cells for CENP-A RNAi with separated SC; *n*= 18 cells for Luc RNAi + nocodazole with joined SC; *n*= 37 cells for CENP-A RNAi + nocodazole with joined SC; *n*= 33 cells for Luc RNAi + MG132 with joined SC; ; *n*= 20 cells for Luc RNAi + MG132 with separated SC ; *n*= 30 cells for CENP-A RNAi + MG132 with joined SC; *n*= 39 cells for CENP-A RNAi + MG132 with separated SC ; **** *P*<0.0001; ** *P*<0.01; * *P*<0.1; N.D.: not determined; nonparametric Kruskal-Wallis test).


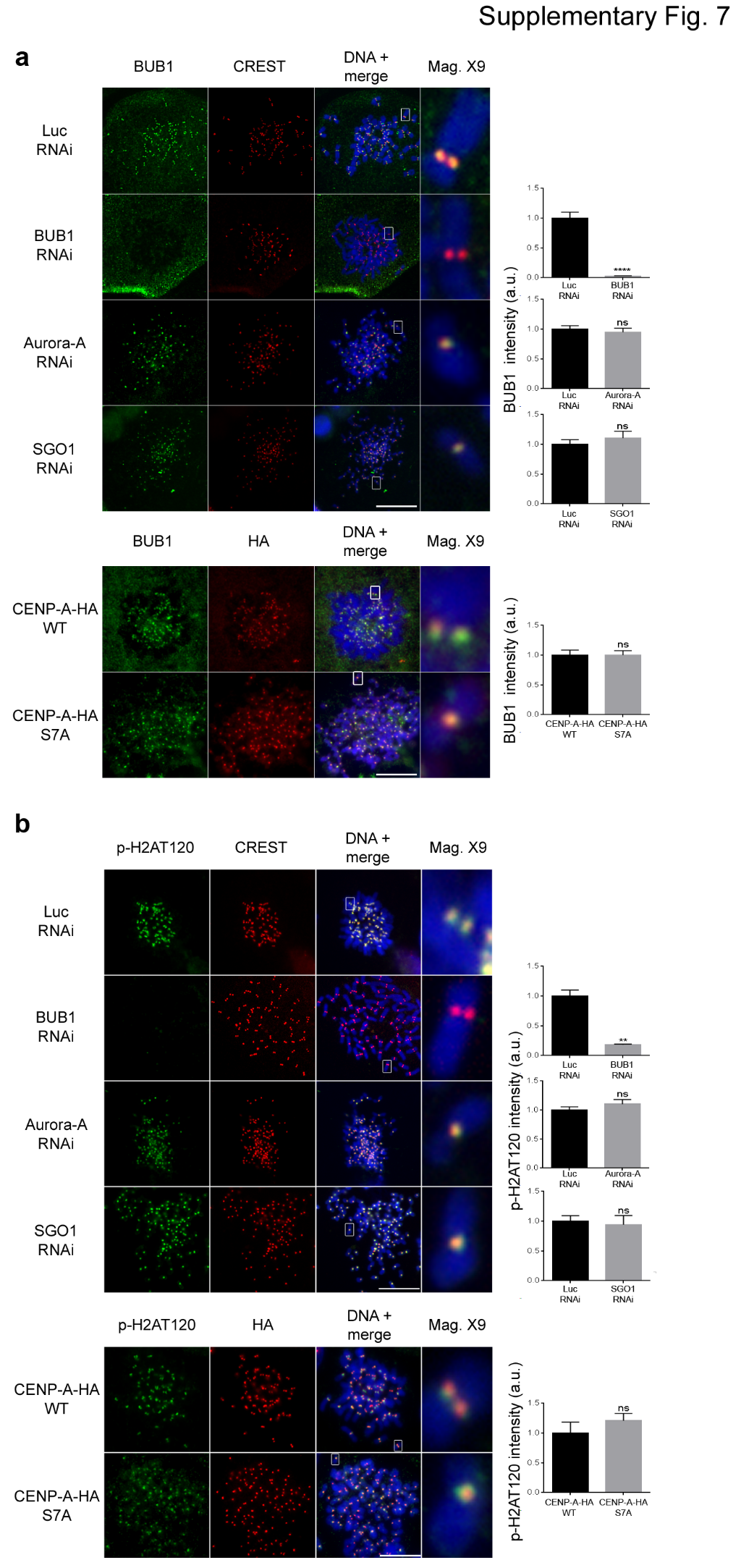


**Supplementary Figure 7**. Metaphase chromosome spreads immunostained with CREST or HA and (A) BUB1, or (B) p-H2AT120 antibodies following the indicated depletions in HeLa-S3 cells or in CENP-A-WT-HA and CENP-A-S7A-HA cell lines. Scale Bar = 5 µm. Single chromosome magnifications are shown. Quantifications of fluorescence intensities for each immunostaining are shown on the right. (means ± SEM; *n* >10 cells; **** *P*<0.0001; *** *P*<0.001; ** *P*<0.01; ns: not significant; nonparametric two-tailed unpaired Mann-Whitney *U* test).


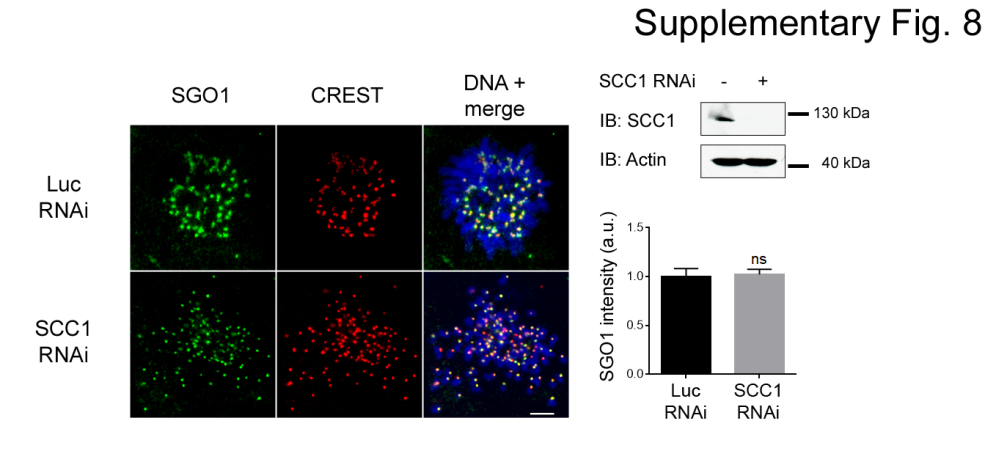


**Supplementary Figure 8**. Metaphase chromosome spreads from Hela S3 cell lines depleted of SCC1 were immunostained with antibodies against Sgo1 and CREST. Scale bar = 5 µm. Quantifications of fluorescence signals for Sgo1 are shown on the right bottom of the panel. The graph shows the means ± SEM from a representative experiment (*n* = 8 cells for Luc RNAi; *n* = 13 cells for SCC1 RNAi; ns: not significant; two-tailed unpaired nonparametric Mann-Whitney *U* test). The corresponding cell extracts were immunoblotted with anti-SCC1 antibody or with anti-Actin antibody as a loading control (right top of the panel).


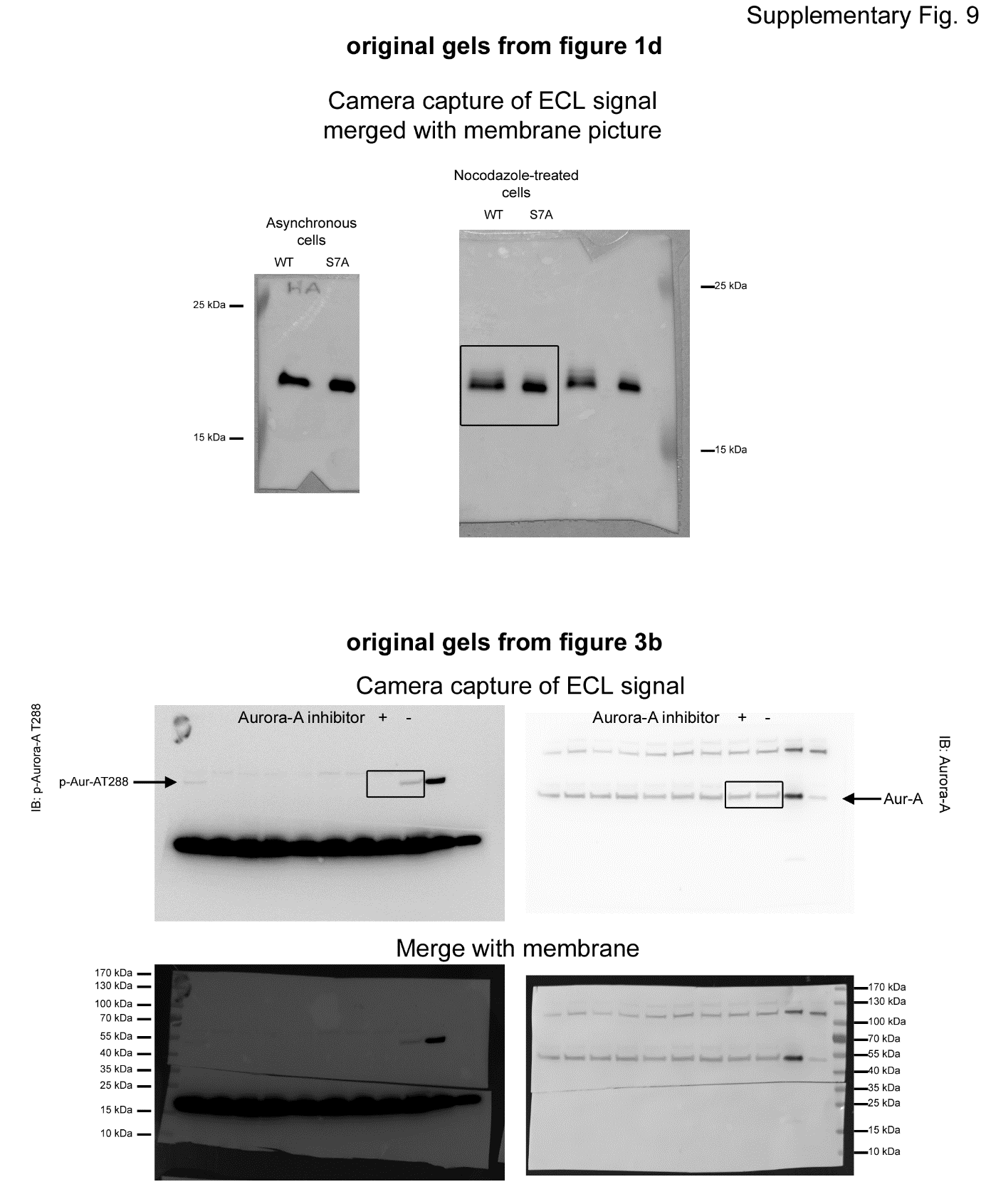


**Supplementary Figure 9.** Original gels scans from figure 1d and 3b


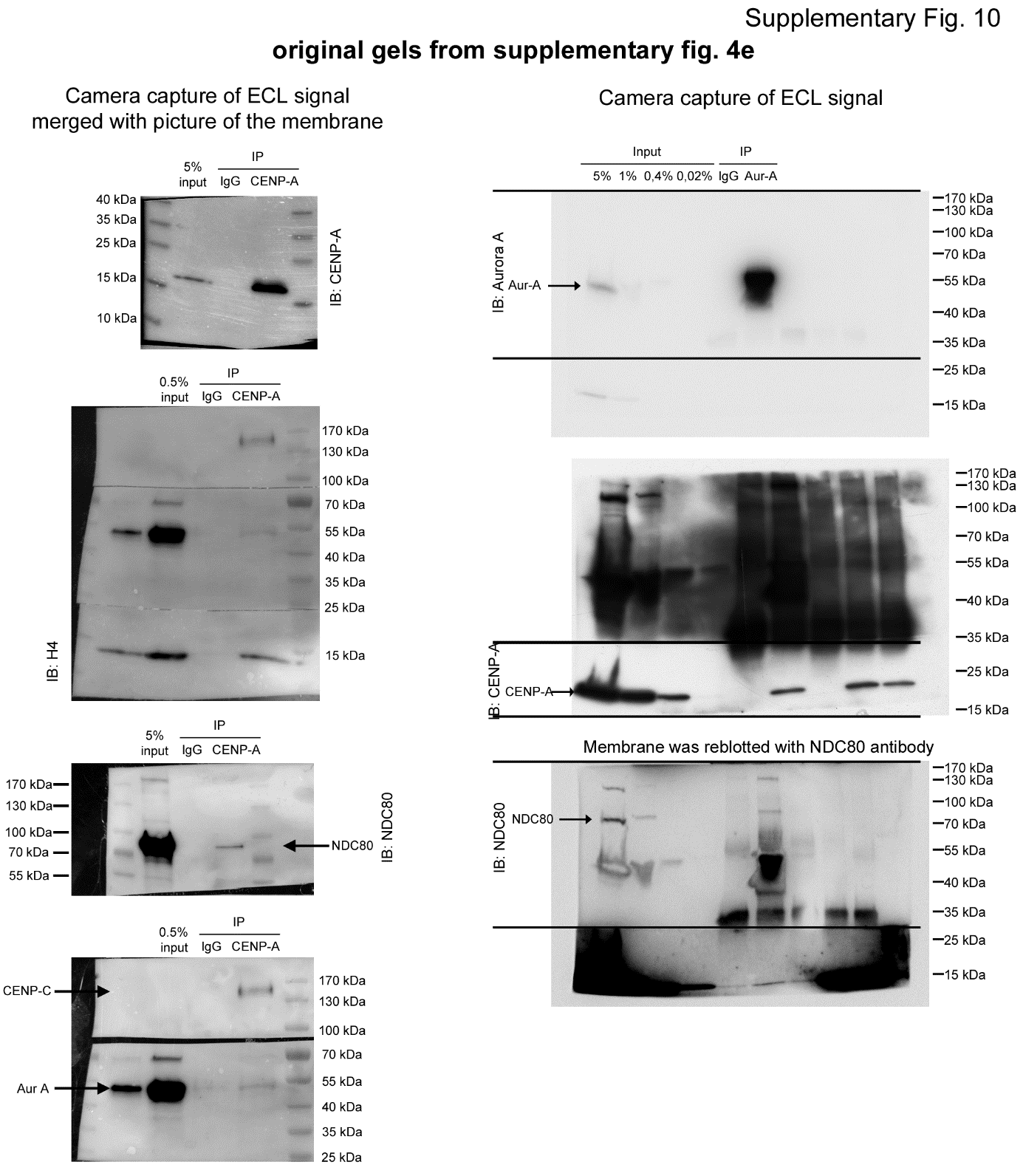


**Supplementary Figure 10.** Original gels scans from supplementary figure 4e
